# Seasonal water use strategies of dominant herbaceous species in a desert steppe revealed by stable isotopes

**DOI:** 10.3389/fpls.2026.1924005

**Published:** 2026-08-19

**Authors:** Bing Zhang, Mingyu Ma, Dongmei Xue, Hai Wang, Fangyu Gao, Xiaoyun Zhang, Stephen M. Mureithi

**Affiliations:** 1 Tianjin Key Laboratory of Water Resources and Environment, Tianjin Normal University, Tianjin, China; 2 Faculty of Geography, Tianjin Normal University, Tianjin, China; 3 Institute of Grassland Research, Chinese Academy of Agricultural Sciences, Hohhot, Inner Mongolia, China; 4 Department of Land Resource Management and Agricultural Technology, University of Nairobi, Nairobi, Kenya

**Keywords:** desert steppe, hydrogen and oxygen stable isotopes, IsoSource, MixSIAR, PS index, water use strategies

## Abstract

**Introduction:**

Global climate change alters the spatiotemporal distribution of soil water, reshaping plant water use strategies in water-limited ecosystems. However, seasonal shifts in plant water-source use and uncertainties associated with different water-source partitioning approaches remain insufficiently quantified in desert steppe ecosystems.

**Methods:**

Using stable hydrogen and oxygen isotopes combined with soil water content (SWC), we investigated the seasonal water sources of *Stipa klemenzii* and *Cleistogenes songorica* in a desert steppe in Inner Mongolia from May to September 2025. Three approaches, the Direct Comparative Method (DCM), IsoSource, and MixSIAR models, were applied to identify and quantify plant water sources.

**Results:**

The results showed that SWC in the 0–5 cm layer exhibited pronounced temporal variability and isotopic enrichment due to evaporation, whereas stability increased with depth. The 20–40 cm layer, constrained by a calcic horizon, served as a stable and isotopically depleted water source during the late growing season. The MixSIAR model exhibited superior performance in quantifying water source contributions compared with the DCM and IsoSource approaches. Both species predominantly utilized water from the 0–10 cm layer during the early and peak growing stages, shifting to water from the 20–40 cm layer when shallow moisture became limited later in the season. Under intensified competition for surface soil water, *C. songorica* increased its reliance on 10–20 cm water, thereby reducing hydrological niche overlap with *S. klemenzii*. The proportional similarity (PS) index peaked in July–August, indicating intensified hydrological niche overlap during the period of maximum water demand.

**Discussion:**

These findings demonstrate depth- and season-dependent water partitioning among dominant herbaceous species in the desert steppe and highlight the importance of Bayesian isotope mixing models for quantifying plant water sources under changing precipitation regimes. The results provide insights into the adaptive water-use strategies of desert steppe plants in response to future climate change.

## Graphical Abstract

**Figure:**
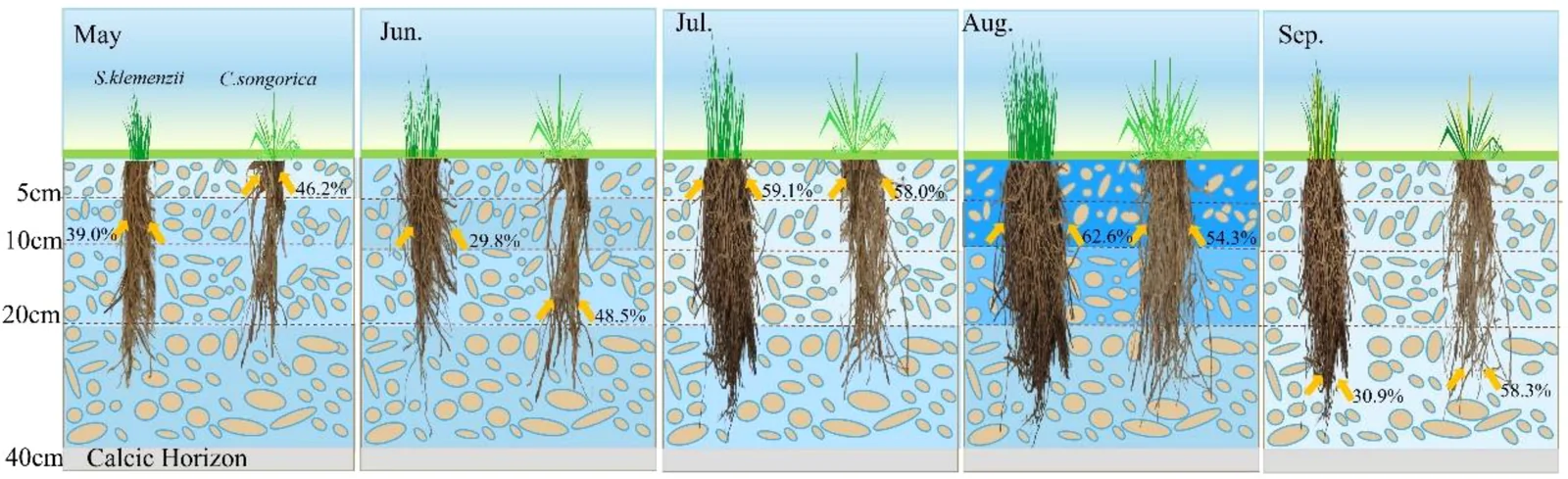
During the growing season from May to September, soil water content peaked in August, and the presence of a calcic horizon restricted the downward infiltration. Plant water sources varied across different growth stages. During the early and peak growing stages, both Stipa klemenzii and Cleistogenes songorica primarily relied on shallow soil water from the 0–10 cm layer. During the peak growing season, the two species relied on similar water sources, resulting in intensified water competition. By the late growing season, both species shifted their water uptake to deeper layers (20–40 cm). During the peak growing season, increased water demand led to substantial overlap in water sources between the two species.

## Highlights

The main novelties of this study are threefold:

- The MixSIAR model exhibited superior performance in quantifying water source contributions compared with the Direct Comparative Method and IsoSource;
- Seasonal shifts from shallow to deep water sources under declining SWC;
- A calcic horizon enhanced water stability and served as an important late-season water source.
- Higher water-source overlap during peak growing season.

## 1 Introduction

Global climate change is accelerating the expansion of arid and semiarid regions and reshaping terrestrial hydrological processes, thereby altering the spatiotemporal dynamics of soil moisture and plant water use patterns (Lei et al., 2023; Sardans et al., 2024). In arid and semiarid ecosystems, soil water is typically the primary source for plant growth, and its variability directly governs plant water use strategies (Heras et al., 2011; Kechen et al., 2022; Kang et al., 2024; Chen et al., 2014). Herbaceous plants, with their shallow root systems, are highly sensitive to fluctuations in soil moisture and can rapidly respond to changes in precipitation regimes (Wang et al., 2019b; Xing et al., 2024). Consequently, increasing frequencies of drought and extreme rainfall events may shift plant water sources, intensify interspecific competition, and threaten community stability (Everingham et al., 2026). Thus, understanding plant water use strategies in arid and semiarid ecosystems is essential for elucidating growth dynamics and responses to environmental change.

Plants of the same species can exhibit seasonal or ontogenetic shifts in water uptake depth, reflecting variability in water availability (Antunes et al., 2018). For example, herbaceous species in the semiarid sandhills of Nebraska primarily rely on shallow soil water but increase their use of deeper sources under drought conditions (Eggemeyer et al., 2009). Similarly, *Vitellaria paradoxa* in West Africa derives approximately 90% of its water from the 10–50 cm layer during the wet season but shifts to deeper soil water and groundwater during the dry season (Bargués Tobella et al., 2017). Through such seasonal or spatial differentiation in water use, plants can reduce direct competition to some extent, thereby maintaining species coexistence. However, under water-limited conditions, coexisting species drawing water from similar soil layers often exhibit high hydrological niche overlap, leading to intensified competition (Schachtschneider and February, 2010). For instance, in the afforestation area on the northeastern edge of the Tengger Desert, *Corethrodendron scoparium* and *Calligonum mongolicum* exhibit reduced water competition during the rainy season when shallow soil moisture is abundant, whereas competition intensifies during the dry season as surface soil moisture becomes depleted (Zhao et al., 2025). Likewise, in the U.S. Midwest Corn Belt, *Symphoricarpos orbiculatus* Moench. and *Quercus alba* L. exhibit a gradual increase in water uptake depth during the growing season as soil water availability declines (Asbjornsen et al., 2008). Water use strategies in the growing season are essential for better understanding mechanisms of adaptation to spatiotemporal water heterogeneity in arid and semiarid grasslands.

Stable hydrogen and oxygen isotope approaches are the most widely used methods for investigating plant water use strategies and ecosystem hydrological processes (Wang et al., 2023; Wu et al., 2022; Silvertown et al., 2014). Since substantial isotopic fractionation generally does not occur during root water uptake, the isotopic signatures of plant xylem water can effectively retain the characteristics of the corresponding water sources (Zhao et al., 2025). The direct comparison method (DCM) was initially used to identify plant water sources by comparing the δD and δ^18^O signatures of xylem water with those of potential water sources (Huo et al., 2018). Furthermore, to quantitatively partition plant water sources under complex multi-source conditions, isotope-based mixing models have been developed to estimate the proportional contributions of different sources, including linear mixing models (e.g., IsoSource) and Bayesian mixing models (e.g., SIAR, MixSIR, and MixSIAR) (Rothfuss and Javaux, 2017). The IsoSource model relies on simple mass-balance calculations without accounting for uncertainties arising from random measurement errors or isotopic fractionation, potentially leading to biased estimates of source contributions (Phillips and Gregg, 2001). However, Bayesian mixing models incorporate prior information and estimate parameters within a probabilistic framework, thereby enabling more robust quantification of the relative contributions of different water sources and their associated uncertainties (Gai et al., 2023). Thus, stable hydrogen and oxygen isotopes integrated with the DCM, IsoSource, and MixSIAR models were widely applied to identify plant water sources.

Sonid Right Banner is located in an arid to semiarid region and represents a typical desert steppe ecosystem characterized by low productivity and high sensitivity to environmental change (Lu et al., 2023). Herbaceous plants generally have shallow root systems and rely primarily on soil water for growth (Zou et al., 2016). However, the mechanisms underlying seasonal shifts in herbaceous plant water use strategies in response to changes in soil water availability remain poorly understood. By integrating stable hydrogen and oxygen isotope analyses with soil moisture measurements, we investigated seasonal variations in water-source use of two dominant herbaceous species (*Stipa klemenzii* and *Cleistogenes songorica*) in a desert steppe ecosystem. The study aimed to: (1) evaluate whether Bayesian mixing models provide more reliable estimates of water source contributions than linear mixing models; (2) identify seasonal shifts in water uptake from shallow to deeper soil layers under declining soil water content; and (3) quantify increased overlap in water-source use between species during the peak growing season. This study provides new insights into the mechanisms underlying how herbaceous plants adapt their water use strategies under seasonal water limitation and advances our understanding of plant–soil water interactions in arid and semiarid ecosystems.

## 2 Materials and methods

### 2.1 Overview of the study area

**Figure 1:**
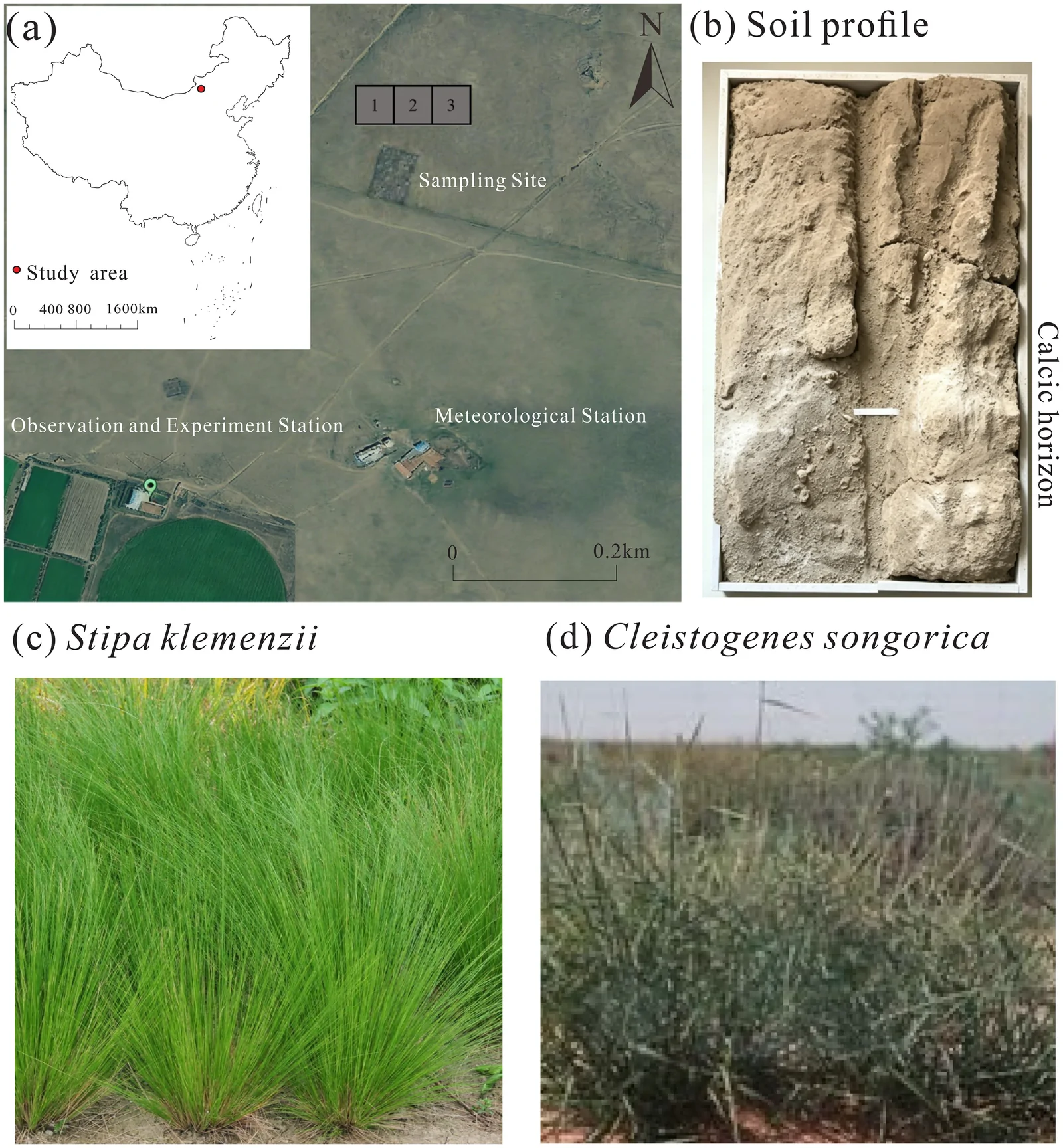
Geographic location of the study area. **(a)** location of the study area in China; **(b)** soil profile; **(c)** *Stipa klemenzii*; **(d)** *Cleistogenes songorica*.

The study area is located at the Sonid Temperate Desert Steppe Resources and Ecological Environment Field Scientific Observation and Research Station of the Chinese Academy of Agricultural Sciences in Xilingol League, Inner Mongolia, China (42°46′52″N, 112°40′25″E) (**Figure 1a**). The area is characterized by a mid-temperate continental climate, with a mean annual air temperature of 4.4 °C. The maximum monthly mean temperature occurs in July (28.1 °C), while the minimum occurs in January (-16.5 °C). Precipitation exhibits a pronounced seasonal pattern, with approximately 50–60% of the annual rainfall occurring in July and August. The average annual precipitation is 177.2 mm, while potential annual evapotranspiration reaches 2384 mm (Fang et al., 2019).

The soil is classified as a typical brown calcareous soil, representing a transitional type from desert to steppe soils, with a calcic horizon commonly occurring at a depth of approximately 40 cm (**Figure 1b**). The study site has been under long-term fencing management, resulting in relatively low anthropogenic disturbance and providing an ideal platform for investigating natural plant water use strategies under semi-natural desert steppe conditions. The vegetation is sparse and species-poor, with an overall vegetation cover of approximately 13% (Shen et al., 2022). The vegetation is primarily dominated by *S. klemenzii* as the dominant species, with *C. songorica* as the associated species (**Figures 1c, d**). *S. klemenzii* exhibits a well-developed fibrous root system characterized by pronounced vertical extension (Yuan et al., 2022). By contrast, *C. songorica* possesses a root system predominantly concentrated in shallow soil horizons but demonstrating marked morphological plasticity, with roots initially distributed in the topsoil and progressively extending into deeper horizons during plant development (Niu, 2017).

### 2.2 Methods

#### 2.2.1 Sample collection

Plant and soil samples were collected on 17 May, 18 June, 17 July, 18 August, and 16 September 2025, with no precipitation recorded during the three days preceding each sampling event. Field sampling was conducted in a long-term fenced grassland within the study area. Three 1 m × 1 m quadrats were randomly established with 20 m spacing between them to ensure independence. Within each quadrat, one healthy individual of *S. klemenzii* and one of *C. songorica* were selected. Samples were collected from the non-photosynthetic tissue at the root-shoot junction, immediately sealed in airtight containers, and stored at 4 °C to prevent evaporative isotopic fractionation prior to laboratory analysis. Soil samples were collected approximately 5 cm away from each sampled plant at four depth intervals (0–5 cm, 5–10 cm, 10–20 cm, and 20–40 cm). For each depth in each plot, two soil cores were taken and composited into a single sample, sealed in airtight bags, and stored at 4 °C for subsequent isotopic analysis.

#### 2.2.2 Water extraction and isotope determination

Water extractions from plant stems and soils, as well as isotopic analyses of samples, were conducted at the Tianjin Key Laboratory of Water Resources and Water Environment, Tianjin Normal University, Tianjin City, China. Water from plant stems and soil samples was extracted using a BJJL-2200 fully automated cryogenic vacuum distillation system (Ehleringer and Dawson, 1992). The SWC was determined by drying method, whereby soil samples were dried to constant weight in the laboratory and weighed to obtain dry mass (Zhao et al., 2025). The SWC was calculated using Equation (1).

(1) $$W=\frac{W_{1}-W_{2}}{W_{2}-W_{0}}\times 100\%$$

In the formula, *W* is the mass water content (%), *W*_0_ is the weight of aluminum box, *W*_1_ is the weight of aluminum box + wet soil, and *W*_2_ is the weight of aluminum box and dried soil.

Hydrogen and oxygen stable isotope ratios (δD and δ^18^O) of water samples were measured using a liquid water isotope analyzer (L2140-i, Picarro, USA) (Zhang et al., 2020a). Isotopic values are reported relative to Vienna Standard Mean Ocean Water (VSMOW), with analytical precisions of ±0.1‰ for δD and ±0.02‰ for δ^18^O. The isotopic values were calculated using Equation (2):

(2) $$\delta =\frac{R_{sample}-R_{VSMOW}}{R_{VSMOW}}\times 1000‰$$

In the formula, *δ* is δD or δ^18^O (‰), *R* is the isotope ratio, $R_{sample}$ is the ratio of heavy and light isotope abundance of elements in the sample (^18^O/^16^O, D/H); $R_{VSMOW}$ is the ratio of stable isotope abundance of international common standard (^18^O/^16^O, D/H stable isotope using VSMOW).

#### 2.2.3 The proportion of potential water sources used by plants

The direct comparison method (DCM), the linear mixing model IsoSource, and the Bayesian mixing model MixSIAR were used to identify seasonal variations in plant water sources of two dominant herbaceous species. The DCM involved directly comparing the stable hydrogen and oxygen isotope compositions (δD and δ^18^O) of plant water with those of soil water from different depths and evaluating isotopic similarity to preliminarily determine potential water uptake depths (Ehleringer and Dawson, 1992).

The linear mixing model IsoSource is applied to estimate the proportional contributions of soil water from different depths to plant water uptake using the feasible range (upper and lower bounds) approach. Because hydrogen isotope fractionation has been reported under certain environmental conditions, particularly in water-limited ecosystems, whereas oxygen isotopes are generally considered to experience less fractionation during plant water uptake, δ^18^O values were selected for IsoSource analysis in this study (Zhao et al., 2024). The IsoSource model is based on isotope mass balance principles and estimates feasible combinations of source contributions under predefined constraints (Phillips and Gregg, 2003). The total contribution of all potential water sources was constrained to 100%, and the feasible contribution ranges for each potential source were determined by setting upper and lower bounds (Phillips and Gregg, 2001). In the model, the δ^18^O value of plant water was used as the mixture signature, while the δ^18^O values of soil water from different depths were assigned as source signatures. The contribution increment was set to 1%, and the tolerance level was set to 0.01. The IsoSource model is based on isotope mass balance principles, as described in Equations (3) and (4):

(3) $$f_{1}\delta^{18}O_{1}+f_{2}\delta^{18}O_{2}+f_{3}\delta^{18}O_{3}+\cdots +f_{n}\delta^{18}O_{n}=\delta^{18}O_{p}$$

(4) $$f_{1}+f_{2}+f_{3}+\cdots +f_{n}=1$$

In the formula, ${\text{δ}}^{18}{\text{O}}_{\text{p}}$ is the oxygen isotope value of extracted plant stem water; ${\text{δ}}^{18}{\text{O}}_{1}$, ${\text{δ}}^{18}{\text{O}}_{2}$, ${\text{δ}}^{18}{\text{O}}_{3}$, …, ${\text{δ}}^{18}{\text{O}}_{\text{n}}$ is the oxygen isotope values of the first, second, third, …, ${\text{n}}_{\text{th}}$ potential water sources, respectively; *f*_1_, *f*_2_, *f*_3_, …, *f_n_* is the proportional contributions of each water source to plant water uptake.

The Bayesian mixing model MixSIAR is used to quantify plant water source contributions by incorporating uncertainty in isotope signatures and source variability. The δD and δ^18^O values of the two plant species were used as mixture inputs. Mean values and standard errors of δD and δ^18^O from different soil layers (0–5, 5–10, 10–20, and 20–40 cm) were used as source inputs. Since no isotopic fractionation occurs during root water uptake by plants (Brooks et al., 2010), discrimination factors were fixed at zero, and both the mean and standard deviation of the discrimination parameters were set to 0 in the MixSIAR model. The Markov Chain Monte Carlo (MCMC) run length was set to “very long.” Posterior distributions of all parameters were examined via Markov chain Monte Carlo (MCMC) simulations to assess model convergence. Convergence diagnostics were deemed prerequisite for subsequent interpretation of MixSIAR outputs. Specifically, model convergence was evaluated using the Gelman–Rubin and Geweke diagnostics (Wang et al., 2017). The error structure was specified as “residual error only,” with uninformative priors applied to all parameters. The MixSIAR model is shown in Equation (5):

(5) $$X_{\;ij}=\sum_{k=1}^{K}P_{k}(S_{jk}+C_{jk})+\epsilon_{ij}$$

In the formula, $X_{ij}$ represents the value of isotope *j* in mixture sample *i*; $S_{jk}$ is the value of isotope *j* in source *k*, assumed to follow a normal distribution with mean $u_{jk}$ and variance $\omega_{jk}^{2}$; $P_{k\;}$ denotes the proportional contribution of source *k* to the mixture; $C_{jk}$ represents the isotopic fractionation factor for isotope *j* in source *k*, assumed to follow a normal distribution with mean $\lambda_{jk}$, and variance $\tau_{jk}^{2}$; $\epsilon_{ij}$ is the residual error term.

#### 2.2.4 Evaluation of model performance

We indirectly evaluated the predictive performance of different water source partitioning methods by assessing the agreement between observed and predicted isotopic compositions of plant water (Wang et al., 2019a). The observed values ( $o_{i}$) were obtained from measured isotopic compositions of plant water collected during the growing season from May to September 2025. Predicted values ( $p_{i}$) were calculated based on the assumption that plant isotopic compositions are mixtures of multiple potential water sources (Stock et al., 2018; Ehleringer and Dawson, 1992). Since IsoSource failed to generate feasible solutions for *C. songorica* in May and June, these samples were excluded from the model performance evaluation. Therefore, the performance metrics for this species were calculated only using samples with valid outputs from both models. The predicted values were calculated using Equation (6):

(6) $$p_{i}=\sum_{j=1}^{k}f_{j}\delta_{A}$$

where *j* denotes the $j_{th}$ water source used by the plants, k is the total number of potential water sources (k = 4 in this study), and *p* represents the proportion contributions of each source to plant water as estimated by water source partitioning methods. $\delta_{A}$ denotes the measured isotopic composition (δD and δ^18^O) of each potential water sources.

To evaluate the performance of different models, five statistical indicators were calculated. The root mean square error was calculated using Equation (7):

(7) RMSE=[1n∑i=1n(pi−oi)2]12

where *n* denotes the number of validation samples. *p_i_* and *o_i_* represent the predicted observed plant water isotopic composition values. Lower *RMSE* values indicate improved model performance with reduced prediction errors.

Goodness-of-prediction statistics (*G*) were also applied to assess model performance. The *G* value of 1 indicated a perfect prediction. Values closer to 1 represent higher model reliability, whereas negative values indicate poor model performance. The G value was calculated using Equation (8):

(8) $$G=1-[\sum_{i=1}^{n}\frac{{\left(p_{i}-o_{i}\right)}^{2}}{\sum_{i=1}^{n}{\left(o_{i}-\bar{o}\right)}^{2}}]$$

where $\bar{o}$ is the average of observed values.

The maximum prediction error (MaxE) and minimum prediction error (MinE) were calculated using Equations (9) and (10):

(9) $$MaxE=\max(p_{i}-o_{i})i=1,\cdots ,\;n$$

(10) $$MinE=\min(p_{i}-o_{i})i=1,\;\cdots ,\;n$$

The model residual error (R) was calculated using Equation (11):

(11) $$R={\left(\delta^{2}H_{t,c}/\delta^{2}H_{t,m}-1\right)}^{2}+{\left(\delta^{18}O_{t,c}/\delta^{18}O_{t,m}-1\right)}^{2}$$

where subscript m refers to the measured values of the isotope mixture, and subscript c represents the values calculated by the mixing models. Smaller *R* values indicated lower model error.

#### 2.2.5 Proportional similarity index

The proportional similarity index is commonly used to quantify niche overlap between coexisting species (Trogisch et al., 2016). A higher PS value indicates greater hydrological niche overlap and stronger competition for the same water sources, whereas a lower *PS* value suggests weaker niche overlap and reduced interspecific competition. The proportional similarity (PS) index was calculated using Equation (12):

(12) $$PS=1-0.5\sum_{i=1}^{\text{n}}|P_{1i}-P_{2i}|$$

In the formula, $P_{1i}$ and $P_{2i}$ represent the utilization ratio of the two plants to the *i* water source, respectively.

#### 2.2.6 Data analysis

One-way analysis of variance (ANOVA) was conducted using IBM SPSS Statistics (version 27.0.1) to assess differences in δD and δ^18^O values of plant and soil water among months. The IsoSource and MixSIAR model was used to analyze the contribution ratios of potential water sources to the plants. Data visualization was conducted using Origin 2024.

## 3 Results

### 3.1 Soil water content

**Figure 2:**
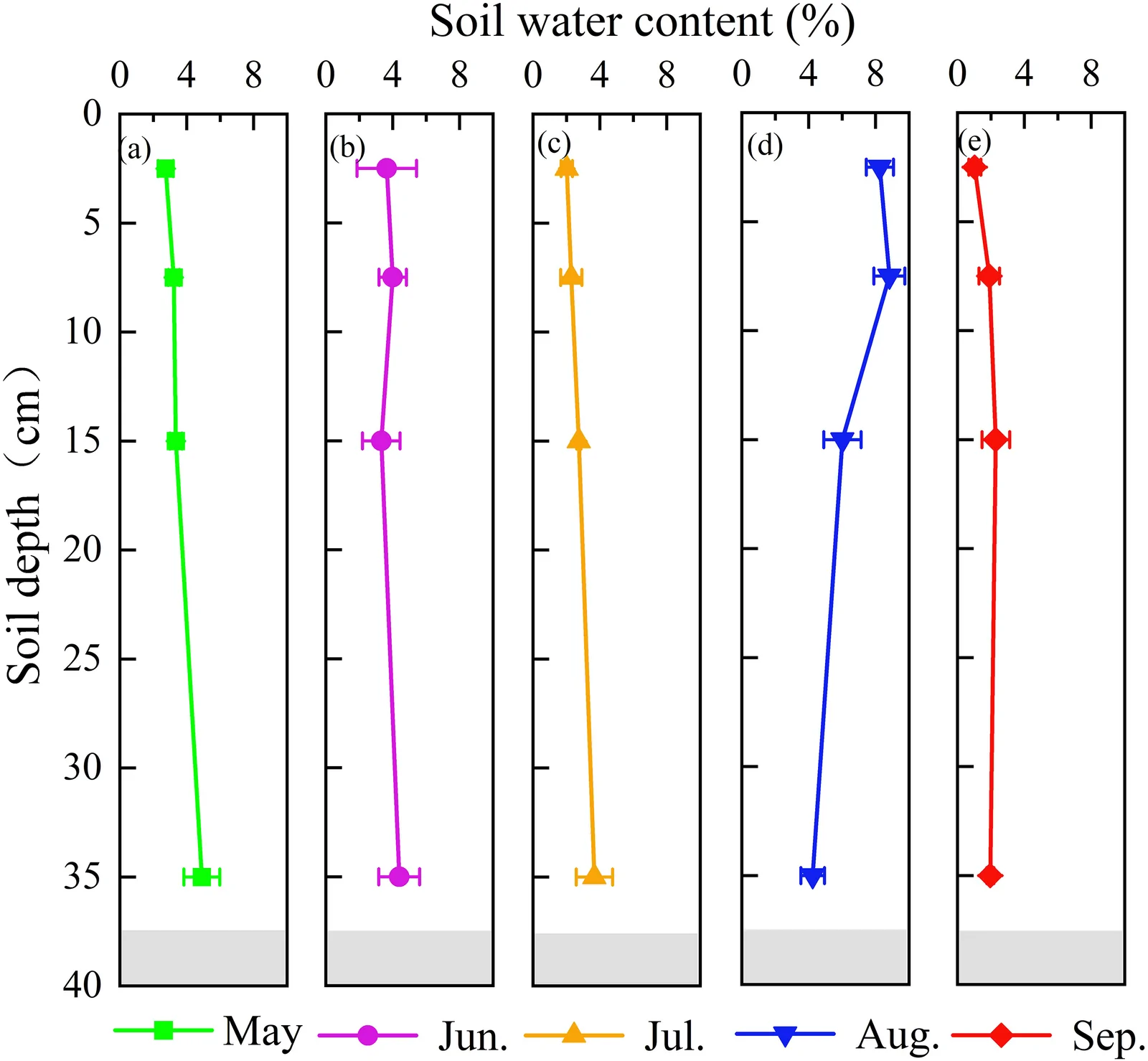
The soil water content of the soil profile during May to September.

Soil water content (SWC) exhibited significant seasonal and vertical variability across the monitoring period (**Figure 2**). SWC reached its maximum in August, significantly exceeding values recorded in other months. Vertical distribution patterns varied markedly among months. In May, SWC increased consistently with soil depth, ranging from 2.74% in the 0–5 cm surface layer to 4.90% in the 20–40 cm subsurface layer (**Figure 2a**). June displayed a more complex vertical profile, with SWC initially increasing, subsequently decreasing, and then increasing again with depth, peaking at 4.39% in the 20–40 cm layer (**Figure 2b**). In July, the SWC consistently increased with depth, and the 20–40 cm layer exhibited significantly higher than the other layers (**Figure 2c**). Notably, August presented a reversed vertical gradient, with SWC initially increasing to a maximum of 8.82% at 5–10 cm depth, then decreasing to a minimum of 4.24% in the 20–40 cm layer (**Figure 2d**). In September, the SWC peaked at 10–20 cm, which was 2.29%, while the lowest value occurred at 0–5 cm, which was 1.03% (**Figure 2e**).

Significant temporal variation in SWC was observed across all soil layers. Except for August, SWC generally increased with soil depth, whereas an inverse vertical pattern occurred in August, likely reflecting intense precipitation inputs. Seasonal fluctuations were most pronounced in the upper 0–20 cm profile: the 0–5 cm, 5–10 cm, and 10–20 cm layers all peaked in August (reaching 8.25%, 8.82%, and 6.02%, respectively) and declined sharply to minimum values in September. In contrast, the 20–40 cm layer exhibited attenuated seasonal dynamics, peaking earlier in May (4.90%) and decreasing progressively to its minimum in September (1.96%), suggesting greater hydrological stability in deeper soil horizons.

### 3.2 Hydrogen and oxygen isotopes in soil water

**Figure 3:**
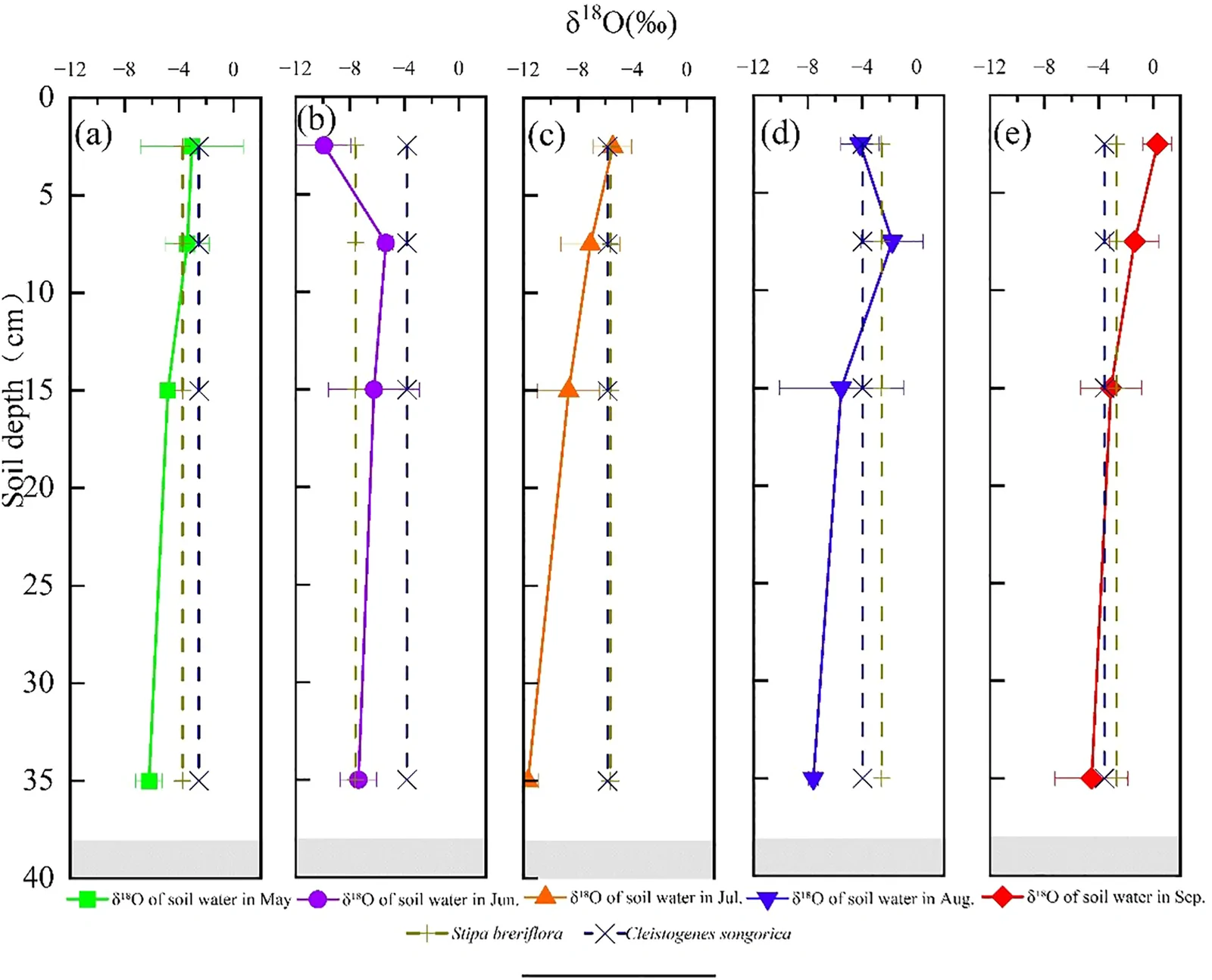
Comparison of δ^18^O values between soil water and plant water across different months. **(a)** May, **(b)** June, **(c)** July, **(d)** August, **(e)** September After the same column of data, different lowercase letters indicate significant differences between different months (p < 0.05).

Soil water δ^18^O values exhibited pronounced temporal fluctuations across different months (**Figure 3** and **Table A.1**). Overall, δ^18^O values decreased fist and then increased with time, reaching their most depleted values in July and becoming most enriched in September. In June, the lowest δ^18^O value occurred in the 0–5 cm soil layer, which was -9.92‰ (**Figure 3b**). In August, the soil water δ^18^O values initially increased and then decreased with soil depth, with the highest value observed in the 5–10 cm layer, which was -3.63‰ and the lowest value in the 20–40 cm layer, which was -6.12‰ (**Figure 3d**).

**Figure 4:**
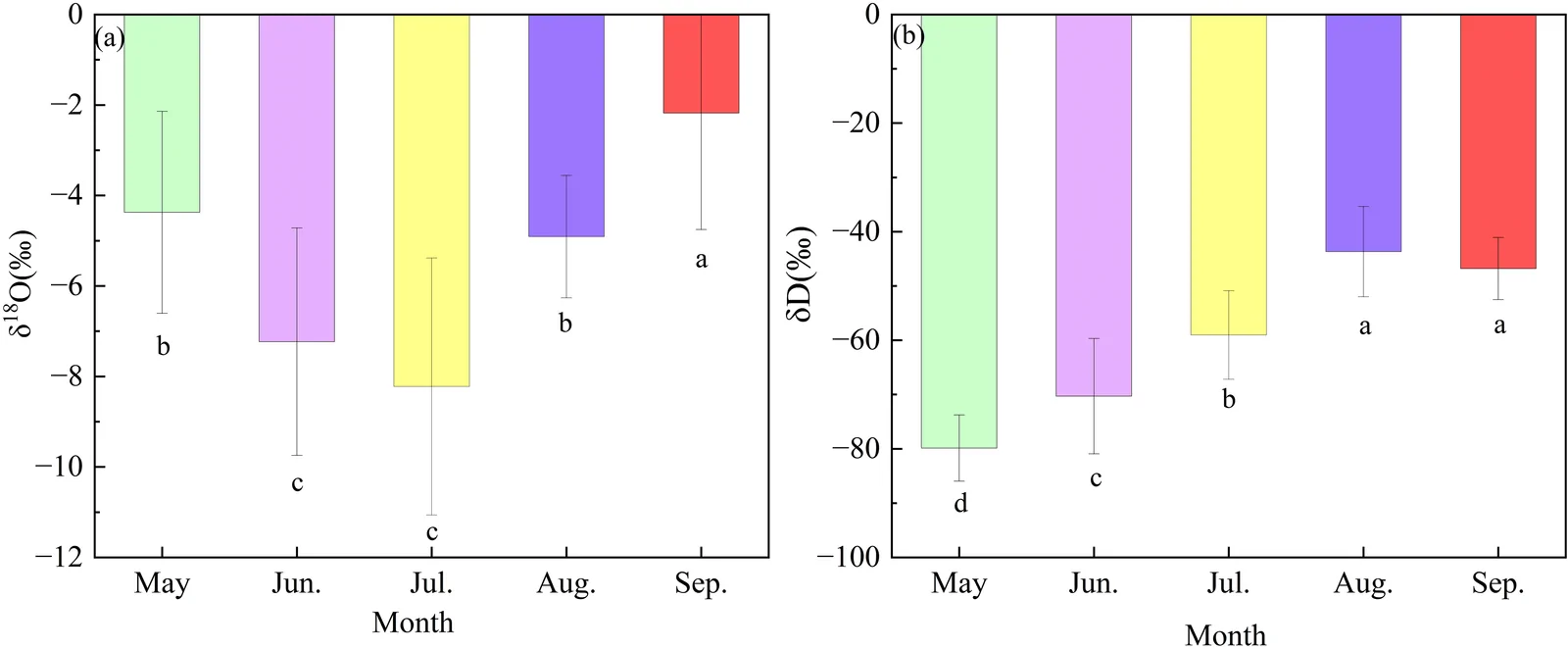
The δD and δ^18^O in soil water during growing seasons **(a)** *S. klemenzii*, **(b)** *C. songorica*.

Soil water δ^18^O and δD values exhibited pronounced seasonal variations (**Figure 4**). After the same column of data, different lowercase letters indicate significant differences between different months (p < 0.05). The δ^18^O values decreased initially and then increased during the growing season, reaching a minimum in July (-8.22‰) and a maximum in September (-2.18‰) (p < 0.05). In contrast, δD exhibited a different seasonal pattern from δ^18^O, with an overall enrichment trend throughout the growing season. The minimum δD value was observed in May (-79.9‰), whereas the maximum occurred in August (-43.7‰) (p < 0.05).

**Figure 5:**
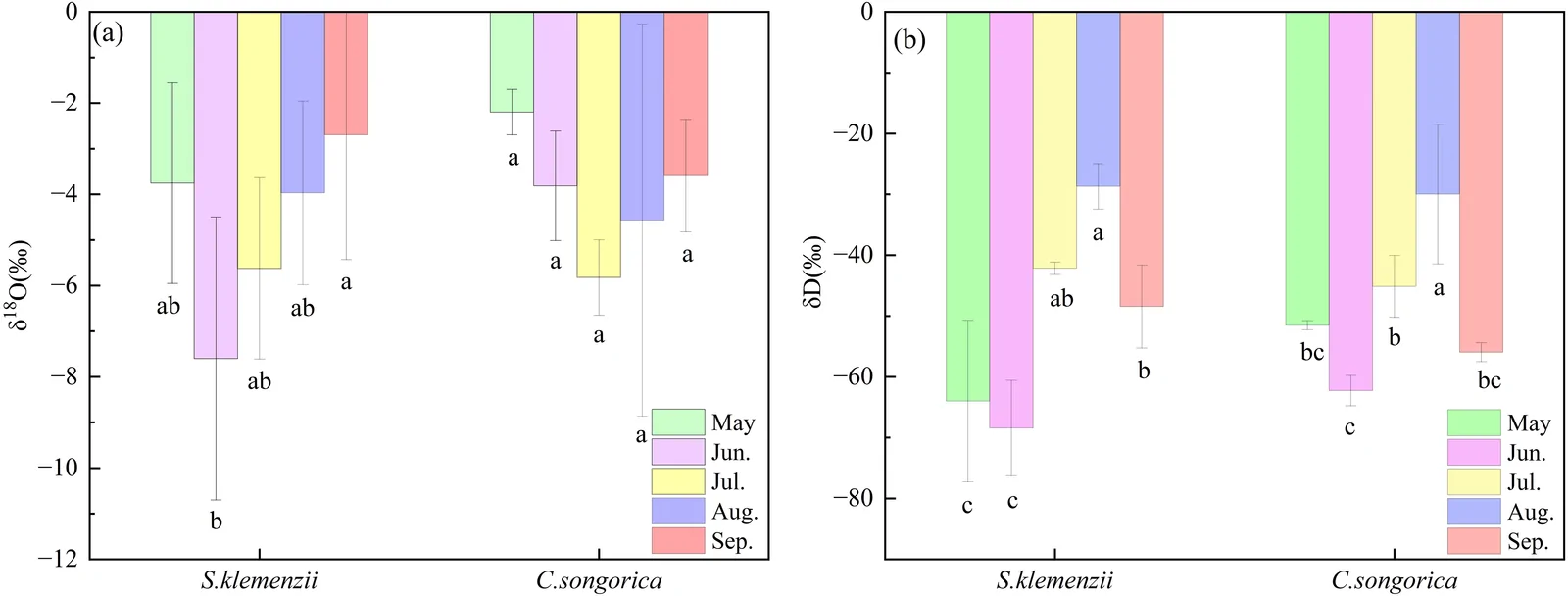
The δD and δ^18^O in water of plants during growing seasons **(a)** *S. klemenzii*; **(b)** *C. songorica*.

Plant water δ^18^O and δD values exhibited pronounced seasonal variations (**Figure 5**). For *S. klemenzii*, the water isotope range was: δ^18^O from -7.60‰ to -2.70‰ and δD from -68.4‰ to -28.7‰; for *C. songorica*, the water isotope range was: δ^18^O from -5.82‰ to -2.55‰ and δD from -62.3‰ to -30.0‰. For *S. klemenzii*, δ^18^O values showed significant differences between June and September (p < 0.05), with the highest values observed in September, which was -2.70‰. For *C. songorica*, δ^18^O values showed not differ significantly among months (p > 0.05), reaching a maximum in May, which was -2.55‰ and a minimum in July, which was -5.82‰. The δD values of *S. klemenzii* and *C. songorica* differed significantly between June and August, with higher values in August and lower values in June.

### 3.3 Qualitative and quantitative analysis of plant water sources

#### 3.3.1 Identification of plant water sources using the DCM

Soil layers whose δ^18^O values intersected with or were closest to those of plant xylem water were identified as the primary water uptake sources (**Figure 3**). In May, the δ^18^O value of *S. klemenzii* water intersected with soil water at only one depth, indicating that soil water from the 5–10 cm layer was its primary potential water source (**Figure 3a**). In June, the intersection occurred at 0–5 cm, suggesting predominant reliance on shallow soil water (**Figure 3b**). In July, the δ^18^O values of water for both *S. klemenzii* and *C. songorica* intersected with soil water at the 0–5 cm layer, indicating that both species primarily utilized shallow soil water (**Figure 3c**). In August, two intersection points were observed between the δ^18^O values of water and soil water for both species, suggesting that soil water from the 0–5 cm and 5–10 cm layers constituted their main water sources (**Figure 3d**). By September, only one intersection was detected for each species: *S. klemenzii* corresponded to the 10–20 cm layer, whereas *C. songorica* aligned with the 20–40 cm layer (**Figure 3e**).

#### 3.3.2 Plant water source contributions estimated using the IsoSource model

**Figure 6:**
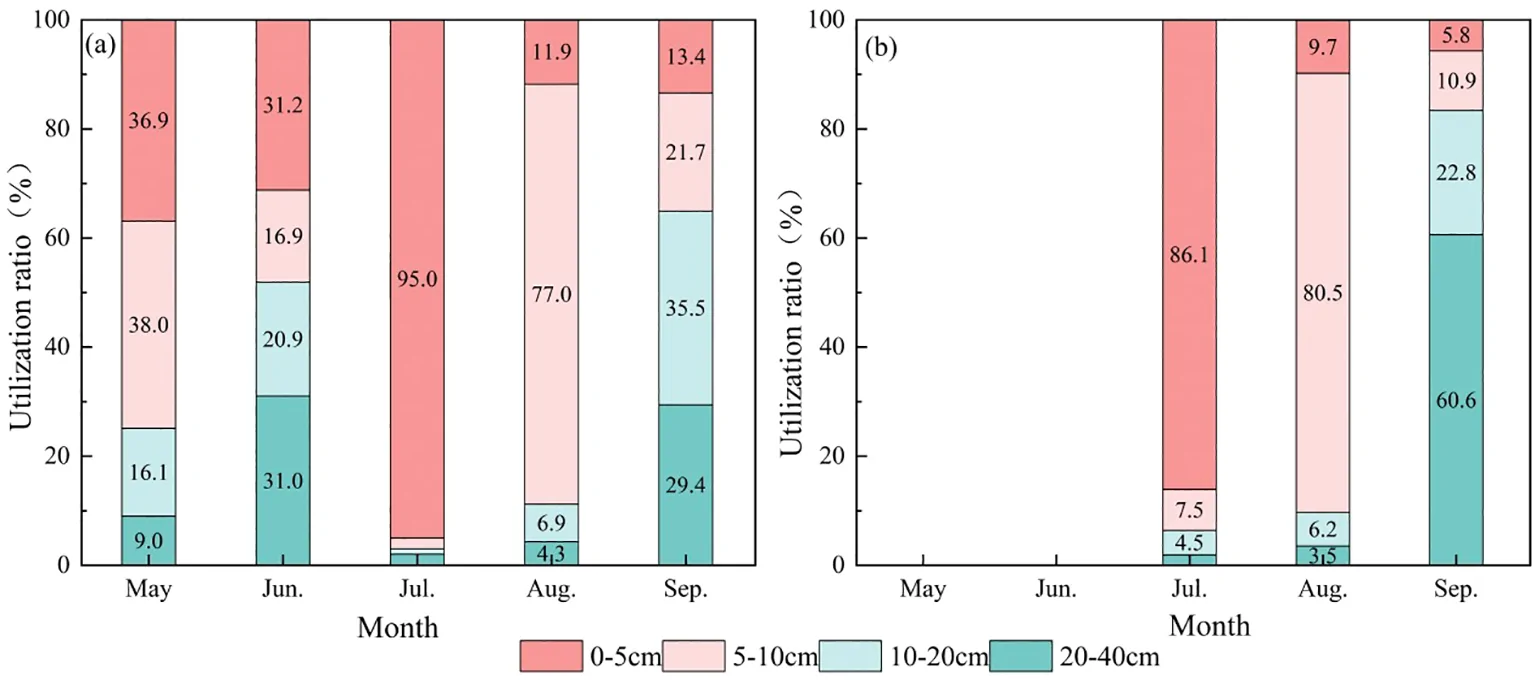
Monthly contributions of potential water sources to plant water uptake as estimated by the IsoSource mixing model. Panels represent **(a)** *S. klemenzii*; **(b)** *C. songorica*.

The results of the IsoSource model showed that the water sources of *S. klemenzii* and *C. songorica* varied with species and seasons (**Figure 6**). For *S. klemenzii*, soil water from the 5–10 cm layer was the primary source in May (38.0%). In June, reliance shifted to the 0–5 cm layer (31.2%), which became the dominant source. In July, shallow soil water (0–5 cm) overwhelmingly dominated water uptake (95.0%). In August, the 5–10 cm layer contributed most (77.0%), whereas in September, water uptake shifted downward to the 10–20 cm layer (35.5%). For *C. songorica*, the 0–5 cm layer contributed 86.1% of total water uptake in July. In August, water derived mainly from the 5–10 cm layer (80.5%), while in September, uptake shifted to deeper soil water (20–40 cm), which accounted for 60.6%.

#### 3.3.3 Plant water source contributions estimated using the MixSIAR model

**Figure 7:**
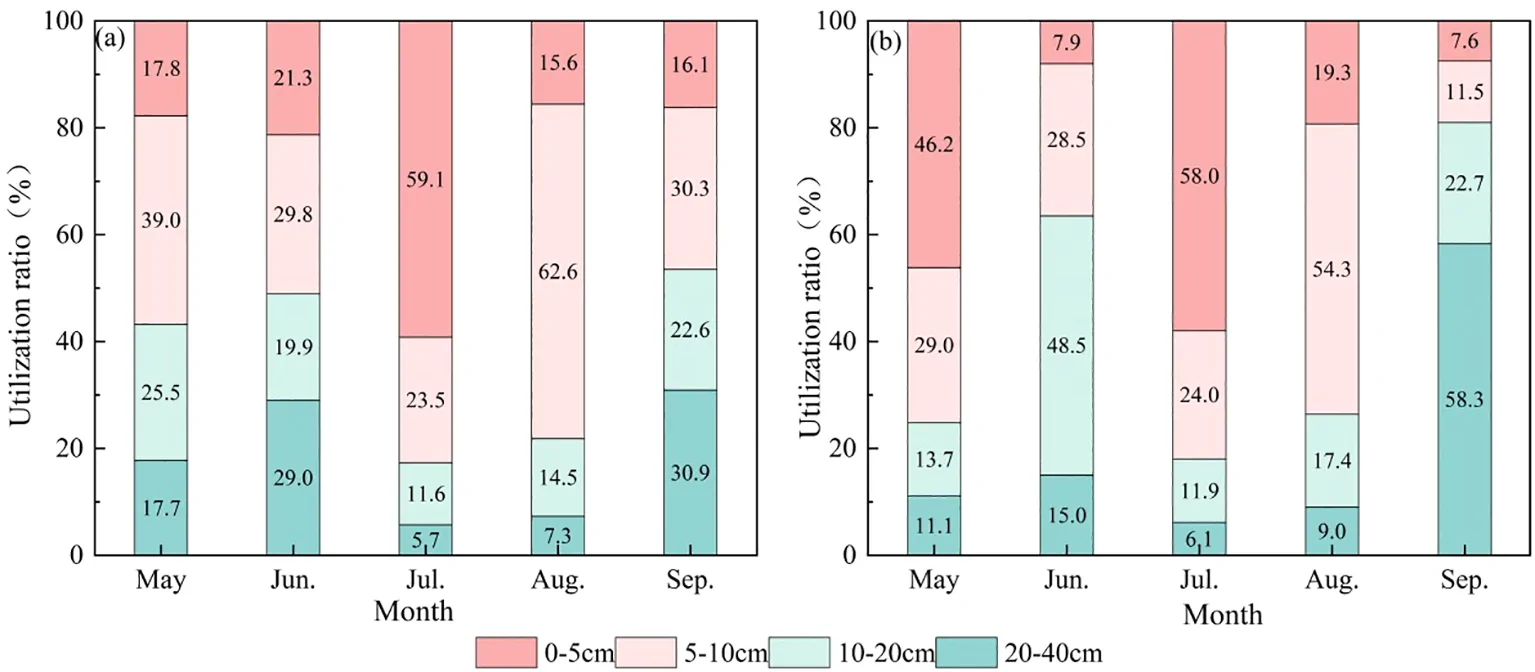
Monthly contributions of potential water sources to plant water uptake as estimated by the MixSIAR mixing model. Panels represent **(a)** *S. klemenzii*; **(b)** *C. songorica*.

The results of the MixSIAR model showed that the water sources of *S. klemenzii* and *C. songorica* varied with species and seasons (**Figure 7**). In May, *S. klemenzii* primarily utilized soil water from the 5–10 cm layer, with a contribution of 39%. In June, *S. klemenzii* continued to rely mainly on the 5–10 cm soil layer. During May and June, water uptake by *C. songorica* was dominated by the 0–5 cm and 10–20 cm soil layers, contributing 46.2% and 48.5%, respectively. In July and August, both *S. klemenzii* and *C. songorica* primarily absorbed water from the 0–5 cm and 5–10 cm soil layers, with contribution rates of 59.1% and 58.0% for *S. klemenzii*, and 62.6% and 54.3% for *C. songorica*, respectively. In September, water uptake by both species shifted predominantly to the 20–40 cm soil layer, accounting for 30.9% and 58.3% of total water uptake for *S. klemenzii* and *C. songorica*, respectively.

#### 3.3.4 Evaluation of model performance

The performance of the three water-source partitioning approaches was comparatively assessed using multiple statistical metrics (**Table 1**). The MixSIAR models showed lower RMSE values than the IsoSource model, indicating higher predictive accuracy. The G-values of IsoSource and MixSIAR were 0.858 and 0.876, respectively, indicating that the models were more reliable. In addition, the parameter R of the MixSIAR model was 0.819, indicating smaller prediction errors in water source partitioning.

**Table 1:** Performance comparison of water source proportion estimates using the IsoSource and MixSIAR models.

Method	RMSE	MaxE	MinE	R	G
IsoSource	1.408	3.815	-0.007	0.860	0.858
MixSIAR	1.069	0.499	-2.636	0.819	0.876

### 3.4 Competition relationship of plant water source

**Figure 8:**
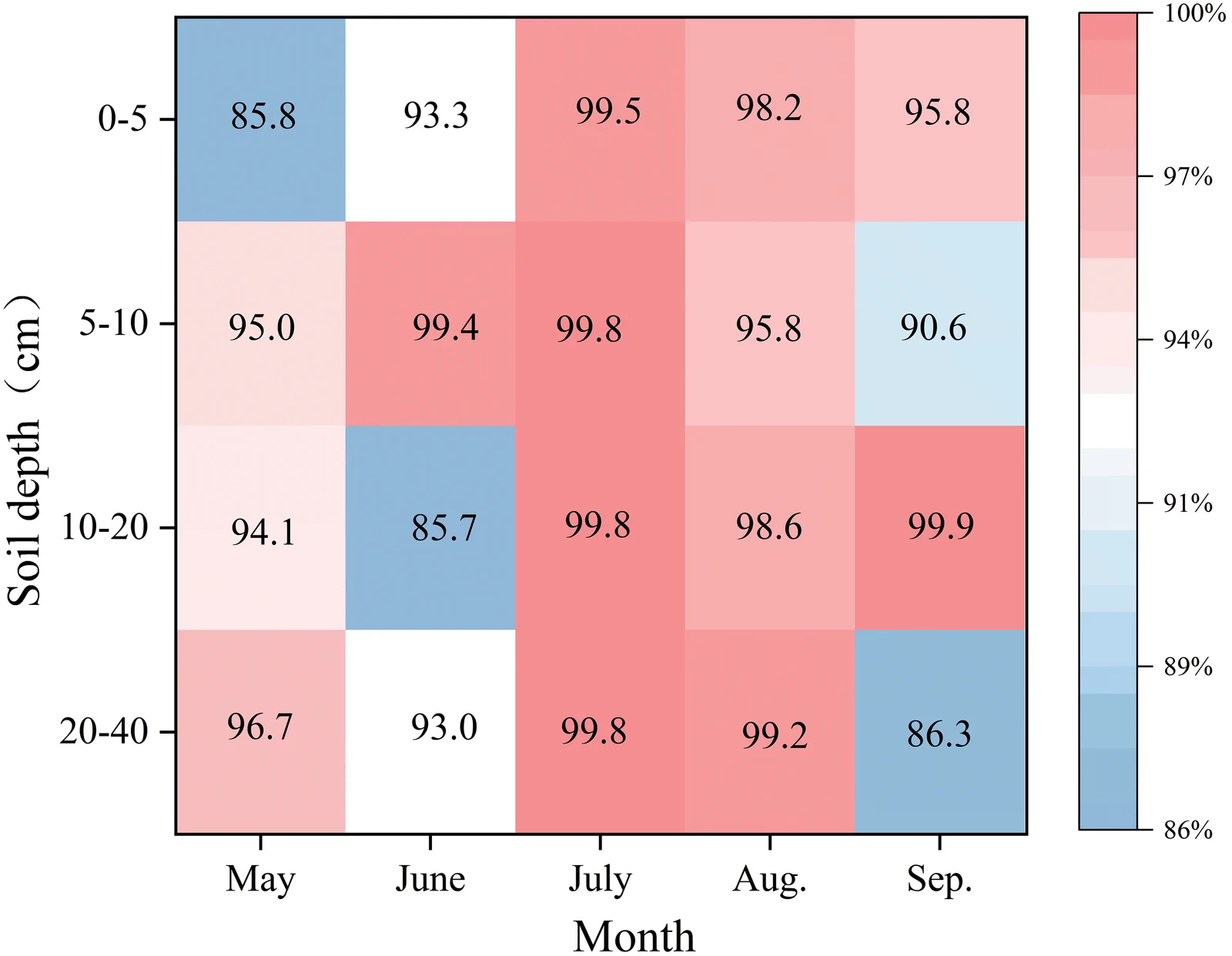
Comparison of PS index of two dominant herbaceous plants. PS, Proportional similarity index; PS = 0 indicates that there is no competition between the two plants. The PS index was between 0–100%, indicating that there was a competitive relationship between the two plants. And the larger the PS index, the stronger the competitive relationship; PS = 100% indicated that the competition between the two species reached the maximum.

The Proportional Similarity (PS) index between *S. klemenzii* and *C. songorica* was generally high, indicating substantial overlap in water sources (**Table A.2**; **Figure 8**). Lower PS values occurred in May, June, and September, suggesting greater niche differentiation and weaker water competition during these months. In contrast, PS values peaked in July (98.8%) and August (91.7%), reflecting strong overlap in shallow soil water use and intensified competition. In September, the PS index reached 99.9% in the 10–20 cm layer, indicating identical water source use at this depth. However, lower PS values were observed in the 0–5 cm layer in May (85.8%), the 10–20 cm layer in June (85.7%), and the 20–40 cm layer in September (86.3%), suggesting reduced competition in these specific layers.

## 4 Discussion

### 4.1 Variations in soil water content and isotopic composition of soil water

Soil water content exhibited pronounced seasonal variability, primarily governed by the combined effects of precipitation and evaporation. In arid and semi-arid regions, precipitation represents the sole source of soil water (Hu et al., 2023) and is mainly concentrated in July and August. The results showed that SWC was relatively low in July, while soil water stable isotope values were more depleted (**Figure 2**). This pattern is mainly attributed to the influence of recent precipitation events with negative isotope values, consistent with findings from the Loess Plateau of China (Wan and Liu, 2016). In August, SWC increased significantly, particularly in the 0–10 cm soil layer, while isotopic differences among soil depths became minimal (**Figures 2**, **3**). This trend reflects substantial soil water recharge from intensive precipitation in July and August, coupled with elevated temperatures that promoted evaporation yet coincided with intensive rainfall that drove infiltration, thereby facilitating effective SWC replenishment (Liu et al., 2024). In contrast, SWC declined sharply in September, concomitant with isotopic enrichment and a reduced slope of the evaporation line, both indicative of intensified evaporative enrichment under limited precipitation input.

Soil water content also exhibited clear vertical stratification, jointly regulated by evaporation and root distribution. The 0–5 cm layer showed low SWC and isotopically enriched values, indicating strong evaporative effects (Geris et al., 2017). With increasing depth, isotopic depletion became more evident, suggesting that evaporative influence weakened downward (**Figures 2**, **3**) (Sprenger et al., 2016). This pattern is consistent with previous findings on vadose-zone water migration in the Qilian Mountains (Yong et al., 2020). Except for August, SWC generally increased with depth, reflecting reduced evaporative loss and plant uptake in deeper layers. The distinct vertical pattern observed in August can be attributed to rainfall pulse effects. Frequent and intense precipitation events rapidly increased SWC in the upper soil layers and enhanced vertical mixing, thereby altering the typical depth-dependent gradient (Yu et al., 2015). Elevated precipitation frequency or intensity markedly enhances SWC in the 0–10cm layer, concurrently attenuating evaporative losses (Liao et al., 2021), thereby sustaining relatively high SWC in shallow layers. Root distribution also contributed to the observed vertical pattern. Previous studies have shown that low root density in deeper soil layers reduces water depletion at depth (Zhao et al., 2025). In this study, SWC variability was greater in the upper 20 cm but became relatively stable at 20–40 cm, indicating that the upper soil layers were more strongly affected by evaporation and root water uptake (**Figure 2**).

The calcic horizon played a key role in regulating deep soil water dynamics. This layer, typically located at approximately 40 cm depth, has low hydraulic conductivity and restricts vertical water movement (Duniway et al., 2010). When precipitation reaches this layer, water movement is impeded, causing accumulation in the overlying soil, allowing the 20–40 cm layer to maintain relatively higher SWC. The 20–40 cm layer further benefits from hydrological isolation: reduced evaporative losses due to vertical separation from the surface, and attenuated plant water uptake due to limited root density within this zone. The relatively higher and more stable SWC in the 20–40 cm layer reflects the formation of a localized perched water zone maintained by the calcic horizon. This observation aligns with previous studies highlighting the water-storage role of calcic horizons during wet periods in Inner Mongolian grassland ecosystems (Li et al., 2013).

### 4.2 Comparative evaluation of the DCM, IsoSource, and MixSIAR model

The inconsistencies in plant water use patterns derived from the DCM, IsoSource, and MixSIAR models mainly reflect differences in their fundamental principles. The DCM identifies plant water sources by directly comparing isotopic signatures between plant and soil water. This approach provides only qualitative results and cannot quantify the proportional contributions of different sources. In addition, when plant isotopic values fall outside the range of potential sources, no intersection occurs, making source identification impossible. This limitation was evident in May and June, when the δ^18^O values of *C. songorica* exceeded the isotopic range of soil water (**Figure 3**).

The IsoSource model estimates source contributions by evaluating all feasible combinations under mass balance constraints. However, it also fails to yield valid solutions when plant isotopic values lie outside the range of source end members (Li et al., 2023), as observed for *C. songorica* in May and June (**Figure 6b**). Even when solutions exist, the software returns only uniform distributions of possible contributions, yielding broad, uninformative solution ranges (Fang et al., 2022) and ignoring spatial heterogeneity in soil or plant isotope fields (Zhang et al., 2020b) (**Figure 2**). For instance, although IsoSource and MixSIAR identified similar potential water sources in July and August, IsoSource estimated slightly higher contribution proportions than MixSIAR (**Figures 6**, **7**). Consequently, IsoSource results may not accurately reflect actual plant water uptake patterns.

In contrast, the MixSIAR model incorporates prior information and explicitly accounts for uncertainty in source variability and measurement error. It generates full posterior probability distributions, enabling more robust and statistically meaningful estimates of source contributions (Wang et al., 2019a; Stock et al., 2018; Parnell et al., 2010). Consequently, MixSIAR provides a more reliable framework for quantifying plant water sources in complex environments. Compared with IsoSource, MixSIAR produced more reliable and stable estimates of plant water sources, as supported by model performance evaluation (**Table 1**). This advantage has also been demonstrated in other semiarid regions, such as the Loess Plateau (Gai et al., 2023; Wang et al., 2019a). MixSIAR is more suitable for assessing the water use strategies of *S. klemenzii* and *C. songorica* in desert steppe ecosystems.

### 4.3 Differences in plant water sources and water use relationship

Both *S. klemenzii* and *C. songorica* exhibited pronounced seasonal shifts in water uptake patterns, reflecting dynamic adjustments in response to SWC variability and root distribution (Kang et al., 2024). During the growing season (May–August), both *S. klemenzii* and *C. songorica* predominantly utilized water from the 0–10 cm soil layer. This pattern is consistent with their root distribution characteristics, as approximately 85% of the root biomass of *S. klemenzii* and about 50% of that of *C. songorica* is concentrated within the 0–10 cm soil layer, enabling efficient use of shallow soil water (Niu, 2017; Yuan et al., 2022).

**Figure 9:**
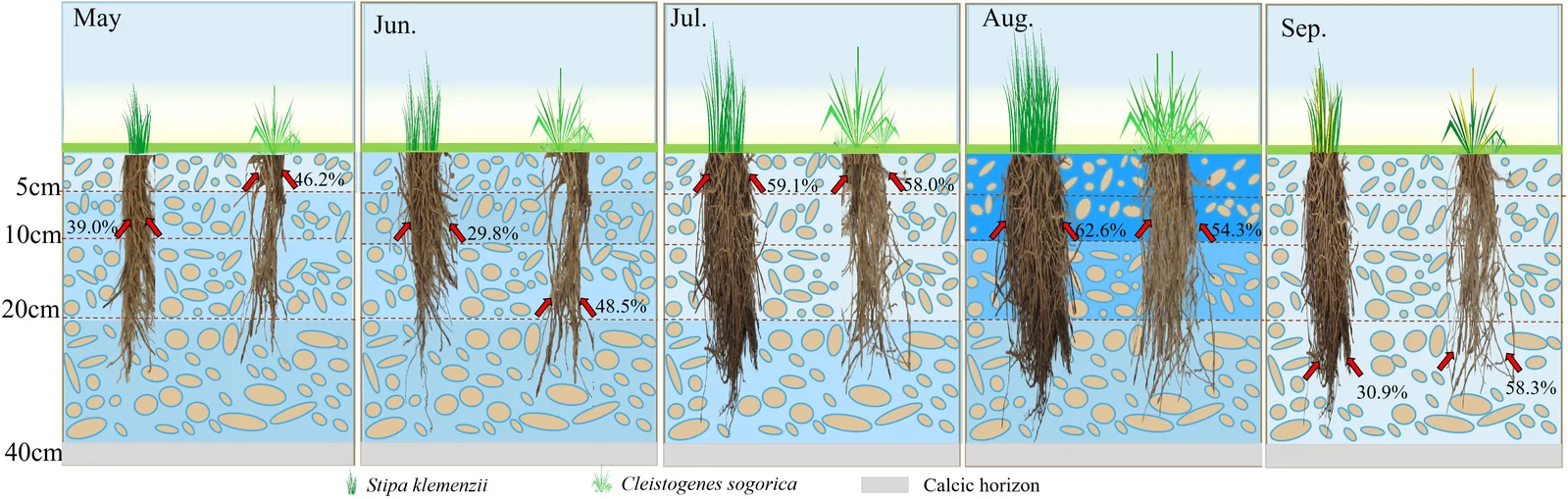
Vertical profiles of plants and soil across different months.

Vertical differences in SWC and root distribution play a critical role in determining plant water uptake depth. In May, both *S. klemenzii* and *C. songorica* primarily relied on soil water from the 0–10 cm layer, likely due to rising surface soil temperatures that enhance shallow root activity (Clarke et al., 2015). *C. songorica* preferentially utilized water from the 0–5 cm layer (**Figures 2**, **7** and **Table A.3**), reflecting both rapid surface SWC response to precipitation pulses and its intrinsic water use strategy (Zhang et al., 2022). Early season precipitation mainly recharges topsoil, resulting in high short-term water availability in the 0–5 cm layer. Although this layer is strongly affected by evaporation, *C. songorica* can rapidly exploit transient water pulses due to its shallow root system concentrated near the surface. In contrast, the 5–10 cm layer is less affected by evaporation and exhibits relatively higher and more stable SWC than the 0–5 cm layer. Consequently, *S. klemenzii* tends to rely on this layer as a relatively stable water source. This divergence suggests niche differentiation in shallow soil water use when near-surface SWC is adequate (Eggemeyer et al., 2009). In June, although SWC in the 5–10 cm layer was relatively high, intense evaporation and elevated root density led to rapid depletion and intensified interspecific competition, thereby reducing effective water availability. Conversely, the 10–20 cm layer exhibited minimal SWC but weaker competition and more stable moisture conditions. Under these conditions, *C. songorica* adjusted its strategy by increasing root length and elongation rates in deeper layers (Niu, 2017), shifting primary water uptake to the 10–20 cm layer. In contrast, *S. klemenzii* maintained reliance on the 5–10 cm layer, where SWC and belowground biomass remained relatively accessible (**Figures 7**, **9**).

During the peak growing season (July–August), water sources of both species exhibited further plastic adjustments. In July, although overall SWC remained relatively low, increased precipitation promoted rapid replenishment of surface moisture. Concurrently, belowground biomass increased significantly in the 0–10 cm layer, while root allocation to the 20–40 cm layer declined (Yang, 2013). This vertical redistribution, combined with rapid shallow SWC response to rainfall pulses, constrained water uptake to the upper profile. Consequently, both species primarily relied on the 0–5 cm layer, where water was most accessible despite high temporal variability. Additionally, elevated nutrient availability in surface soils stimulated root proliferation and reinforced shallow uptake (Nippert and Knapp, 2007). In August, frequent precipitation significantly increased SWC across all layers, with maximum values in the 5–10 cm layer. Concurrently, belowground biomass in the upper 10 cm layer reached its seasonal maximum (Yang, 2013), enhancing root water uptake capacity. Compared with surface soil, the 5–10 cm layer experienced weaker evaporative losses and maintained higher and more persistent moisture, providing favorable conditions for plant uptake (Yu et al., 2012). Consequently, both species shifted their primary water source from the dynamic uppermost 5 cm to the relatively stable and water-abundant 5–10 cm layer. This pattern aligns with previous evidence that herbaceous species in desert steppe ecosystems predominantly exploit shallow soil water during periods of high precipitation inputs (Hu et al., 2021).

Plant water uptake in September was primarily regulated by shallow soil water limitation and hydrological redistribution associated with the calcic horizon. By September, the late stage of the growing season, SWC had declined across all layers relative to earlier months. The 0–5 cm layer experienced severe evaporative depletion, exhibiting the lowest SWC and the most enriched stable isotope signatures. In contrast, the calcic horizon restricted vertical infiltration and promoted water retention in the overlying soil, allowing the 20–40 cm layer to maintain relatively higher and more stable SWC. Concurrently, evaporative losses at this depth were greatly reduced due to its distance from the soil surface. Under these conditions, shallow soil water became insufficient to meet plant water demand. Consequently, both species shifted water acquisition to the deeper and more stable 20–40 cm layer (**Figures 2**, **7**). This shift suggests that when shallow soil water becomes limited, plants can adjust their water uptake depth to utilize relatively stable deeper soil water, thereby maintaining physiological activity and growth. Such a transition from shallow to deeper water sources is considered an important adaptive strategy for plants coping with water stress in arid and semiarid ecosystems, which is consistent with findings from the Konza Prairie Biological Station (Nippert and Knapp, 2007).

### 4.4 Competition relationship of plant water source

High PS index values across most of the growing season reveal extensive hydrological niche overlap between *S. klemenzii* and *C. songorica*, which is consistent with findings from desert steppe ecosystems in northern China (Hao et al., 2024). This pattern primarily reflects their convergent root distribution strategies. Both species are shallow-rooted herbs, with most roots concentrated within the upper 10 cm layer, leading to strong reliance on shallow soil water during the growing season. However, hydrological niche overlap exhibited clear temporal dynamics in response to SWC availability and competition intensity.

The PS index peaked in July and August, coinciding with frequent precipitation pulses that rapidly increased SWC in the upper 10 cm layer. In August, elevated shallow SWC combined with peak root biomass in the upper 10 cm layer enhanced water uptake capacity. Under conditions of abundant water supply and dense root distribution, both species preferentially exploited shallow soil water, intensifying competition and resulting in greater niche overlap. In contrast, lower PS values in May, June, and September indicated reduced competition and enhanced niche differentiation. In June, despite moderate SWC, strong evaporation and high root density accelerated depletion of shallow soil water, increasing competition intensity. Under these conditions, *C. songorica* shifted its water uptake to the 10–20 cm layer by increasing root length density in the 10–30 cm soil profile (Niu, 2017), thereby reducing competition with *S. klemenzii* in the shallow layer (**Figure 9**). By September, intensified evaporation further depleted shallow SWC, while the presence of a calcic horizon restricted deep percolation and maintained relatively stable SWC in the 20–40 cm layer. Consequently, both species shifted water uptake to the 20–40 cm layer, effectively alleviating competition for shallow soil water (**Table A.2**).

Similar patterns of dynamic water partitioning have been reported in other arid and semiarid ecosystems. For example, in semiarid sand dune grasslands of the United States, herbaceous and woody plants shift water sources in response to soil moisture dynamics (Eggemeyer et al., 2009). In northern China, *Pinus tabuliformis* and *Hippophae rhamnoides* exhibit a progressive shift from shallow to deeper soil water with decreasing precipitation (Tang et al., 2018). Collectively, these findings demonstrate that the dynamic regulation of water uptake depth, driven by precipitation variability, SWC redistribution, and root plasticity, is a key ecological mechanism that reduces competition and facilitates species coexistence in water-limited ecosystems.

### 4.5 Limitations and perspectives

This study provides insights into seasonal variations in plant water use strategies, focusing exclusively on the growing season. However, the monthly sampling frequency may have limited our ability to capture short-term changes in plant water use, particularly following episodic precipitation events. Future shifts in precipitation regimes, including reduced light rainfall events, increased heavy precipitation, and prolonged drought periods, may further influence soil water dynamics and plant water use strategies (Li et al., 2013; Qian et al., 2010). As freeze–thaw processes may alter soil water redistribution, infiltration, and availability in the area, which may influence soil water distribution (Wang et al., 2019c). Moreover, root traits were inferred from previous studies rather than directly measured at the study site (Schenk and Jackson, 2002). Future studies integrating high-frequency isotope monitoring, direct measurements of root traits, and precipitation manipulation experiments will further improve our understanding on the hydrological process of the soil-plant-atmosphere continuum (SPAC) system under changing climatic conditions.

## 5 Conclusions

The stable isotopes (δD and δ^18^O), soil water content measurements, and water-source partitioning approaches, including the direct comparison method (DCM), IsoSource, and MixSIAR models, were applied to characterize seasonal water use strategies of two dominant herbaceous species, *S. klemenzii* and *C. songorica*, in a desert steppe ecosystem of Inner Mongolia. Soil water availability exhibited pronounced seasonal and vertical variations. The 0–5 cm soil layer showed strong fluctuations associated with precipitation inputs and evaporative enrichment, whereas the 20–40 cm soil layer maintained relatively stable moisture conditions, likely due to the buffering effect of the calcic horizon, and served as an important water source during periods of limited surface moisture availability. Comparison of water-source partitioning approaches demonstrated that MixSIAR provided more robust estimates of source contributions by incorporating uncertainty, highlighting the importance of selecting appropriate models for interpreting plant water use strategies under complex multi-source conditions.

Herbaceous species exhibited flexible water acquisition strategies by dynamically adjusting their reliance on different soil water sources in response to seasonal changes in water availability. During May–August, both species primarily utilized water from the 0–10 cm soil layer. As shallow soil water content declined in September, both species increased their reliance on water from the 20–40 cm soil layer. Compared with *S. klemenzii*, *C. songorica* exhibited greater flexibility in adjusting water-source utilization, with increased reliance on the 10–20 cm soil layer under conditions of limited surface soil moisture availability. Seasonal changes in water-source overlap further indicated that hydrological niche interactions between the two species were closely associated with seasonal variations in plant water demand and soil water availability. The PS index reached 98.8% and 91.7% in July and August, respectively, indicating the greatest water-source overlap during the peak growing season, whereas lower overlap during the early and late growing stages suggested stronger hydrological niche differentiation. These findings suggest that dominant herbaceous species in desert steppe ecosystems may promote coexistence through flexible adjustment of water uptake depth and seasonal partitioning of soil water resources.

The interpretation of plant water use strategies remains subject to several uncertainties, primarily due to the lack of non-growing season observations and the limited temporal resolution of isotope sampling, which restricted our ability to detect rapid plant water-use responses following individual precipitation events. Future studies integrating high-frequency isotope monitoring, *in situ* assessments of root architectural and functional traits, and controlled precipitation manipulation experiments will further improve our understanding of plant water use strategies under changing climatic conditions. Such integrated approaches will further enhance our understanding of soil–plant–atmosphere water interactions, particularly in water-limited arid and semiarid ecosystems.

## Data Availability

The original contributions presented in the study are included in the article/**Supplementary Material**. Further inquiries can be directed to the corresponding author.
