# Supplementary material for: Seasonal water use strategies of dominant herbaceous species in a desert steppe revealed by stable isotopes
Source: Front Plant Sci. 2026 Aug 19;17:1924005. doi: 10.3389/fpls.2026.1924005 (PMC13533971; doi:10.3389/fpls.2026.1924005)
Supplement: Supplementary file 1 [file DataSheet1.docx]

***Supplementary material***

Table A. 1 presents the soil water content and hydrogen and oxygen isotope values at different depths from May to September. Both soil water content and stable hydrogen and oxygen isotopes exhibited dynamic fluctuations across seasons and depths. Among them, August showed the highest water content in all soil layers along with the most depleted stable hydrogen and oxygen isotopes, whereas September exhibited the lowest water content and the most enriched stable isotopes across all depths.

Table A. 1 Variation characteristics of soil water content and hydrogen and oxygen isotopes

| Month | soil depth (cm) | N | SWC (%) | soil water | |
| --- | --- | --- | --- | --- | --- |
|  |  |  |  | δ^18^O (‰） | δD (‰） |
| May | 0–5 | 3 | 2.79±0.48 | -3.06±3.78 | -81.6±7.8 |
|  | 5–10 | 3 | 3.21±0.35 | -3.39±1.63 | -76.5±5.6 |
|  | 10–20 | 3 | 3.18±0.37 | -4.83±0.34 | -80.7±7.3 |
|  | 20–40 | 3 | 4.10±1.36 | -6.21±0.97 | -80.7±5.9 |
| June | 0–5 | 3 | 3.23±1.71 | -9.92±1.99 | -83.8±4.1 |
|  | 5–10 | 3 | 3.74±1.02 | -5.38±0.50 | -66.2±4.3 |
|  | 10–20 | 3 | 3.19±1.13 | -6.24±3.35 | -60.9±9.5 |
|  | 20–40 | 3 | 3.79±1.35 | -7.39±1.34 | -70.4±8.0 |
| July | 0–5 | 3 | 2.02±0.34 | -5.44±1.41 | -52.3±6.4 |
|  | 5–10 | 3 | 2.28±0.63 | -7.08±2.17 | -54.8±6.5 |
|  | 10–20 | 3 | 2.74±0.12 | -8.69±2.30 | -60.5±6.8 |
|  | 20–40 | 3 | 3.67±1.09 | -11.68±0.75 | -68.5±2.0 |
| August | 0–5 | 3 | 6.44±3.23 | -4.65±0.60 | -39.1±1.4 |
|  | 5–10 | 3 | 7.05±3.60 | -3.63±0.97 | -36.2±5.4 |
|  | 10–20 | 3 | 5.19±2.15 | -5.24±1.96 | -44.9±6.9 |
|  | 20–40 | 3 | 3.78±0.97 | -6.12±0.16 | -54.5±3.1 |
| September | 0–5 | 3 | 1.62±1.23 | 0.30±1.04 | -40.0±2.8 |
|  | 5–10 | 3 | 2.27±0.88 | -1.37±1.82 | -46.7±1.5 |
|  | 10–20 | 3 | 2.41±0.92 | -3.11±2.25 | -46.9±4.5 |
|  | 20–40 | 3 | 2.31±0.63 | -4.54±2.67 | -53.6±3.0 |

Fig. A.1 and A.2 present the quantified proportional contributions of water sources for *Stipa klemenzii* and *Cleistogenes songorica* at different soil depths across months, as determined by the IsoSource model. The results indicate that in May, the solution for *S. klemenzii* converged within the 5–10 cm soil layer, suggesting a predominant reliance on water from this depth. Convergence occurred in the 0–5 cm layer in June and July, shifted back to the 5–10 cm layer in August, and moved to the 10–20 cm layer in September. For *C. songorica*, convergence was observed in the 0–5 cm layer in July, the 5–10 cm layer in August, and the 20–40 cm layer in September.

Fig. A.1 Histograms obtained by the multi-source mass balance, which shows the estimated ranges of proportional contribution of water from each soil depth to total water uptake of *Stipa klemenzii*

Fig. A.2 Histograms obtained by the multi-source mass balance, which shows the estimated ranges of proportional contribution of water from each soil depth to total water uptake of *Cleistogenes songorica*.

Table A.2 shows the monthly variation in the proportional similarity (PS) index of water use between *S. klemenzii* and *C. songorica*. Lower PS values in May, June, and September correspond to divergent water-use patterns and weaker competition between the two species. In contrast, the higher PS values observed in July and August indicate intensified competition for water resources during these months.

Table A.2 Monthly variation in the proportional similarity (PS) index between two plant species.

| Time | PS index of two plants |
| --- | --- |
| May | 71.60 |
| June | 71.35 |
| July | 98.85 |
| August | 91.70 |
| September | 72.60 |

Table A.3 presents a comparison of the water sources for *Stipa klemenzii* and *Cleistogenes songorica* as determined by three different methods. The results indicate consistent water uptake depths for *S. klemenzii* across all three methods in May, July, and August. Similarly, for *C. songorica*, the three methods yielded congruent findings regarding its water sources in July.

Table A.3 Comparison of plant water sources using three methods.

| Month | *Stipa klemenzii* | | | | *Cleistogenes songorica* | | | | |
| --- | --- | --- | --- | --- | --- | --- | --- | --- | --- |
|  | the direct comparison | IsoSource model | MixSIAR model | the direct comparison | | IsoSource model | | MixSIAR model | |
| May | 5–10cm | 5–10cm | 5–10cm | | 0–5cm | |  | | 0–5cm |
| June | 0–5cm | 0–5cm | 5–10cm | | 5–10cm | |  | | 10–20cm |
| July | 0–5cm | 0–5cm | 0–5cm | | 0–5cm | | 0–5cm | | 0–5cm |
| August | 5–10cm | 5–10cm | 5–10cm | | 0–5cm | | 5–10cm | | 5–10cm |
| September | 10–20cm | 10–20cm | 20–40cm | | 10–20cm | | 20–40cm | | 20–40cm |

In May, the MixSIAR model indicated that *S. klemenzii* primarily relied on shallow soil water sources, with the highest posterior median contribution originating from the 5–10 cm soil layer.

Fig. A.3 Posterior distributions of water source contributions to *S. klemenzii* in May based on the MixSIAR model.

In May, the MixSIAR model indicated that *S. klemenzii* primarily relied on shallow soil water sources, with the highest posterior median contribution originating from the 5–10 cm soil layer.

Fig. A.4 Posterior distributions of water source contributions to *C. songorica* in May based on the MixSIAR model.

In June, the MixSIAR model indicated that *S. klemenzii* primarily relied on shallow soil water sources, with the highest posterior median contribution originating from the 0–5 cm soil layer.

Fig. A.5 Posterior distributions of water source contributions to *S. klemenzii* in May based on the MixSIAR model.

In June, the MixSIAR model indicated that *C. songorica* primarily relied on soil water from the 10–20 cm layer, which showed the highest posterior median contribution.

Fig. A.6 Posterior distributions of water source contributions to *C. songorica* in June based on the MixSIAR model.

In July, the MixSIAR model indicated that *S. klemenzii* and *C. songorica* primarily relied on soil water from the 0–5 cm layer, which showed the highest posterior median contribution.

Fig. A.7 Posterior distributions of water source contributions to *S. klemenzii* in July based on the MixSIAR model.

Fig. A.8 Posterior distributions of water source contributions to *C. songorica* in July based on the MixSIAR model.

In August, the MixSIAR model indicated that *S. klemenzii* and *C. songorica* primarily relied on soil water from the 5–10 cm layer, which showed the highest posterior median contribution.

Fig. A.9 Posterior distributions of water source contributions to *S. klemenzii* in August based on the MixSIAR model.

Fig. A.10 Posterior distributions of water source contributions to *C. songorica* in August based on the MixSIAR model.

In September, the MixSIAR model indicated that *S. klemenzii* and *C. songorica* primarily relied on soil water from the 20–40 cm layer, which showed the highest posterior median contribution.

Fig. A.11 Posterior distributions of water source contributions to *S. klemenzii* in September based on the MixSIAR model.

Fig. A.12 Posterior distributions of water source contributions to *C. songorica* in September based on the MixSIAR model.
